# Metta-based group meditation and individual cognitive behavioral therapy (MeCBT) for chronic depression: study protocol for a randomized controlled trial

**DOI:** 10.1186/s13063-019-3815-4

**Published:** 2020-01-06

**Authors:** Artjom Frick, Isabel Thinnes, Ulrich Stangier

**Affiliations:** Institute for Psychology - Department of Clinical Psychology and Psychotherapy, Goethe University Frankfurt, Varrentrappstr. 40-42, 60486 Frankfurt am Main, Germany

**Keywords:** Metta, Loving kindness meditation, Mindfulness, Cognitive behavioral therapy, Chronic depression, Persistent depressive disorder, Randomized controlled trial

## Abstract

**Background:**

Depression is a widespread disorder with severe impacts for individuals and society, especially in its chronic form. Current treatment approaches for persistent depression have focused primarily on reducing negative affect and have paid little attention to promoting positive affect. Previous studies have shown that metta meditation increases positive affect in chronically depressed patients. Results from previous trials provide evidence for the efficacy of a stand-alone metta meditation group treatment in combination with mindfulness-based approaches. Further research is needed to better understand the implementation of meditation practice into everyday life. Therefore, mindfulness and metta meditation in a group setting are combined with individual cognitive behavioral therapy (CBT) into a new, low-intensity, cost-effective treatment (“MeCBT”) for chronic depression.

**Methods/design:**

In this single-center, randomized, observer-blinded, parallel-group clinical trial we will test the efficacy of MeCBT in reducing depression compared to a wait-list control condition. Forty-eight participants in a balanced design will be allocated randomly to a treatment group or a wait-list control group. Metta-based group meditation will be offered in eight weekly sessions and one additional half-day retreat. Subsequent individual CBT will be conducted in eight fortnightly sessions. Outcome measures will be assessed at four time points: before intervention (T0); after group meditation (T1); after individual CBT (T2); and, in the treated group only, at 6-month follow-up (T3). Changes in depressive symptoms (clinician rating), assessed with the Quick Inventory of Depressive Symptoms (QIDS-C) are the primary outcome. We expect a significant decline of depressive symptoms at T2 compared to the wait-list control group. Secondary outcome measures include self-rated depression, mindfulness, benevolence, rumination, emotion regulation, social connectedness, social functioning, as well as behavioral and cognitive avoidance. We will explore changes at T1 and T2 in all these secondary outcome variables.

**Discussion:**

To our knowledge this is the first study to combine a group program focusing on Metta meditation with state-of-the art individual CBT specifically tailored to chronic depression. Implications for further refinement and examination of the treatment program are discussed.

**Trial registration:**

ISRCTN, ISRCTN97264476. Registered 29 March 2018 (applied on 14 December 2017)—retrospectively registered.

## Background

### Chronic depression: epidemiology and treatment approaches

Major depression is a widespread [1] disorder that involves significant psychosocial impairment [2]. If untreated, major depression frequently takes a recurrent or chronic course [3] and is associated with high economic burden [4]. A high proportion of the affected population suffers persistently from depressed mood, loss of interest, and other symptoms of depression, with some fluctuations but no distinct episodes [5]. The lifetime prevalence for chronic forms of depression is about 2–5% of the population in western countries [6, 7]. Chronic forms of depression are associated with significantly greater social and health-related impairment, increased comorbidity rates, and higher risk of suicide than less persistent forms of depression [8, 9]. Due to their homogeneous nature, the four subtypes of chronic depression (cf. Keller et al. [10]) were combined into one diagnosis in the Diagnostic and Statistical Manual of Mental Disorders, Fifth Edition (DSM-5): Persistent depressive disorder (PDD) [11].

Despite the heavy burden of long-lasting symptoms, chronic forms of depression are often not recognized and are not treated sufficiently and adequately according to national guidelines [12]. In general, success rates and treatment effects on patients with chronic depression are significantly lower than for non-chronic forms of depression [13]. The results of a network meta-analysis [14] that included pharmacotherapy, interpersonal psychotherapy (IPT), the Cognitive Behavioral Analysis System of Psychotherapy (CBASP), and combinations of pharmacotherapy and psychotherapy indicated effectiveness of medication, CBASP, and a combination of IPT and medication. A more recent meta-analysis [15] found moderate effect sizes of CBASP compared to treatment as usual (TAU) and IPT, small effect sizes against other psychological treatments, and no significant difference between CBASP and pharmacotherapy.

A possible alternative to psychotherapy approaches that focus on interpersonal processes is the combination of cognitive therapy with mindfulness-based meditation (MBCT) [16]. This has been shown to successfully reduce relapse in recurrent depression [17], with effects similar to pharmacological treatment [18]. Evidence for a possible efficacy of MBCT in chronic depression comes from non-randomized trials [19–22]. On the other hand, a recent randomized controlled study concluded that rather active, problem-focused interventions such as CBASP may be more effective than passive, self-related interventions such as MBCT [23]. One reason for the worse performance of MBCT could be the neglect of fostering positive emotions. However, the results of that study must be interpreted with caution as there were different outcomes for MBCT at the two sites of the study and MBCT was conducted in unusually small groups (four to six participants) compared to more traditional group sizes (eight to twelve participants).

### The role of positive affect in chronic depression

Research on the role of affect in depression implies that positive affect and negative affect should be regarded as distinct and independent factors [24]. Depression has been characterized as a state of high negative affect and low positive affect [25]. This implies that in the treatment of depression negative affect must be reduced while promoting positive affect. In chronic depression, experiential avoidance [26] and rumination are especially related to negative affect and have a strong impact on the persistence of depression [27]. Mindfulness meditation may be a good approach to reduce these mechanisms, as it fosters a de-identifying stance towards negative—but also positive—appraisal. However, there is still a need for patients to engage in activities that promote positive affect.

Fredrickson has formulated an empirically supported theory (*broaden and build-theory*) on why the increase of positive affect can be beneficial for individuals in the long term and how this can be promoted [28]. According to her theory, people who experience positive emotions *broaden* their attention and are better able to think flexibly, as opposed to negative emotions, where the attentional focus is often narrowed to threats. When positive emotions occur more often, and perception and thinking have been broadened, the possibility of *building* personal resources is created. Our approach extends current treatment approaches by focusing on correlates of positive affect on a more basic level, namely the attitude of benevolence and the readiness and ability to experience positive emotions.

### Metta meditation as a treatment for mental disorders

In Buddhism, “metta” (Pali; “benevolence”, “loving-kindness”, “friendliness”) is one of the four essential “attitudes”. It refers to a mental state of unselfish and unconditional kindness to all beings that one develops through meditation and cultivation in relations with others [29]. The main Western psychological and philosophical concept that refers to metta is benevolence, a value or attitude which refers to the motivation to do good. Unlike mindfulness-based meditation, metta meditation (or loving-kindness meditation) explicitly aims to promote positive emotional states and a positive attitude towards oneself and others [29]. By concentrating on individually derived repeated formulas, the motivation for positive activities, benevolent behavior, and the improvement of relations with other people is encouraged [30–32]. PDD is often associated with high self-criticism. A greater motivation to be more benevolent towards oneself may help to compensate for this deficit [33, 34]. In addition, the enhancement of positive affect may also contribute to the increase of resilience [35]. Furthermore, people suffering from PDD often have problems in their interpersonal relationships, such as a rather more submissive and hostile interpersonal style [36].

A recently published meta-analysis based on 22 controlled studies, most of which were conducted in the general population [37], showed that metta meditation not only reduces depression but also improves psychological well-being and interpersonal relationships. In two within-subjects single-arm pilot studies with patients suffering from PDD, our working group found evidence supporting the feasibility and safety of a group treatment that combined mindfulness and metta meditation with cognitive-behavioral techniques. In the first pilot study (*N* = 12) we found moderate to large effect sizes, based on depressive symptoms, after completion of treatment and at 3-month follow-up [38]. The patients reported significant changes in their emotion regulation strategies: suppression of negative feelings and rumination decreased, while acceptance increased. In a subsequent, also uncontrolled study (*N* = 12), a modified meditation program was evaluated [35]. After completion of treatment, large effects were achieved in the decrease in depressive symptoms. In addition, participants reported a significant increase in the ability to tolerate negative emotions. However, in both pilot studies the samples were small, control conditions were missing, and no longer term follow-up data were collected. Evaluations of qualitative interviews with patients after the treatment highlighted their difficulties in implementing the emotion-regulation techniques targeted in meditation into daily life activities. For example, a large proportion of patients with PDD show more experiential avoidance [39] and withdrawal from previous social life [40] compared to patients with recurrent depression. Therefore, we developed the intervention further to include subsequent individual sessions using cognitive-behavioral techniques to overcome problems in daily practice of meditation and to translate its goals, i.e., benevolence towards oneself and others, into concrete action.

### Overcoming avoidance as a potential mechanism in the treatment of chronic depression

Avoidance and suppression are dysfunctional strategies to regulate negative emotions [41] as they prevent recovery from depressive episodes [42]. In turn, improving an accepting attitude towards negative emotions is a significant predictor of successful treatment not only in MBCT [43] but also in CBT [44]. Interestingly, there is growing evidence that people with depression suppress not only negative and burdensome emotions but also positive emotions [45]. The avoidance of positive emotions may further reduce the motivation for engaging in activities that are positively reinforcing. A chronic history of depression was also linked to stronger tendencies to self-reported behavioral and cognitive/emotional avoidance than an episodic history of depression [46]. Thus, avoidance on a behavioral level as well as experiential avoidance may explain the maintenance and chronicity of depression. Meta-analyses showed that traditional CBT, and in particular behavioral activation [47], was superior to control conditions as well as medical treatment [48]. Thus, we chose to include CBT behavioral activation as well as cognitive restructuring as treatment components that may counteract avoidance tendencies.

### Childhood trauma and persistent chronic depression

A large body of research [49–52] indicates that childhood trauma or childhood adversity are risk factors for the development of PDD. Chronic depression was also significantly associated with a greater lifetime traumatic load and a higher prevalence of PTSD [7]. In a recent German study with *N* = 349 chronically depressed patients [53], 76% of the participants reported clinically relevant histories of traumatic experiences in childhood on at least one scale of the Childhood Trauma Questionnaire (CTQ). In this study the multiplicity of traumas in childhood was the strongest predictor of symptom severity, with emotional abuse and sexual abuse being the strongest predictors. This is consistent with findings that patients with a more persistent rather than episodic course of depression were significantly more likely to have suffered from childhood emotional abuse or neglect [39]. Differences in the individual history of childhood maltreatment in patients with PDD may influence the individual etiology and require special treatment elements. There is evidence that schema therapy may be effective in treating dysfunctional cognitive, emotional, and behavioral patterns due to early negative experiences [54]. Schema therapy is largely overlapping with CBT, including techniques of cognitive restructuring and behavioral activation, but adds interventions that focus on processing early maladaptive experiences and enhancement of healthy adult functioning. Therefore, to overcome stressful memories of adverse childhood experiences and the resulting dysfunctional schemas, we will also incorporate techniques of schema therapy as an option into our treatment.

### Rationale for the current trial

Based on the effects of metta meditation on positive motivation and affects, as well as the findings on deficits in chronic depression related to behavioral and emotional avoidance and early maladaptive schemas, we combined group metta meditation with individual CBT (including behavioral activation and schema therapy) to reduce depression as the principal aim of the treatment. As secondary outcomes of the study, we will assess variables that deliver information about the effects of essential strategies included in the treatment protocol:

1. Improving emotion regulation, increasing acceptance of negative thoughts, and reducing rumination by mindfulness meditation
2. Increasing benevolence and motivation to improve relations with other people
3. Overcoming avoidance

The effects of the treatment on primary and secondary outcomes will be compared with changes in a wait-list control group in a single-center, randomized, observer-blinded, parallel-group study design.

### Hypotheses and research questions

The primary outcome measure in this trial is the difference in the severity of depressive symptoms between the two groups. The main research question is: Does the combination of group meditation and individual CBT lead to significantly greater reduction in depressive symptoms compared to a wait-list control condition after overall completion of treatment (at T2)?

The previous data suggest that, at least in the short term, a stabilization of mood improvements is possible by maintaining a regular meditation practice [38]. So far, little research has focused on the maintenance of therapy success of group meditation programs for PDD. Therefore, a secondary question is: Does the effect in the treated group appear to be maintained at the 6-month follow-up?

In addition to the primary outcome measure, further changes will be recorded regarding specific effect parameters related to the two treatment components (meditation and CBT). The aim is to enable an intermediate measurement to identify changes in the two phases of treatment. Therefore, another secondary question is: Compared to control group, are there any significant changes in patients’ mindfulness, benevolence, rumination, emotion regulation, social connectedness, social motivation, and behavioral and cognitive avoidance (a) after the group meditation program and (b) after completion of the overall treatment?

The design of this study does not allow us to test the separate effects of both components, group meditation and individual CBT, which would require a different design. Furthermore, we will not be able to conduct adequate mediation analyses as the statistical power would not be sufficient to do so. However, exploratory analyses will be conducted to examine whether pre-post changes in depression in the group meditation program are associated with changes in the subsequent individual treatment.

In addition, in line with research indicating that mindfulness-based meditation is associated with changes in emotion regulation, especially acceptance and revaluing [32], and the reduction of avoidance [55], we will explore whether changes in the variables “emotion regulation”, “behavioral avoidance”, and “cognitive avoidance” are associated with the primary outcome at post-treatment.

## Methods/design

### Design and setting

**Fig. 1:**
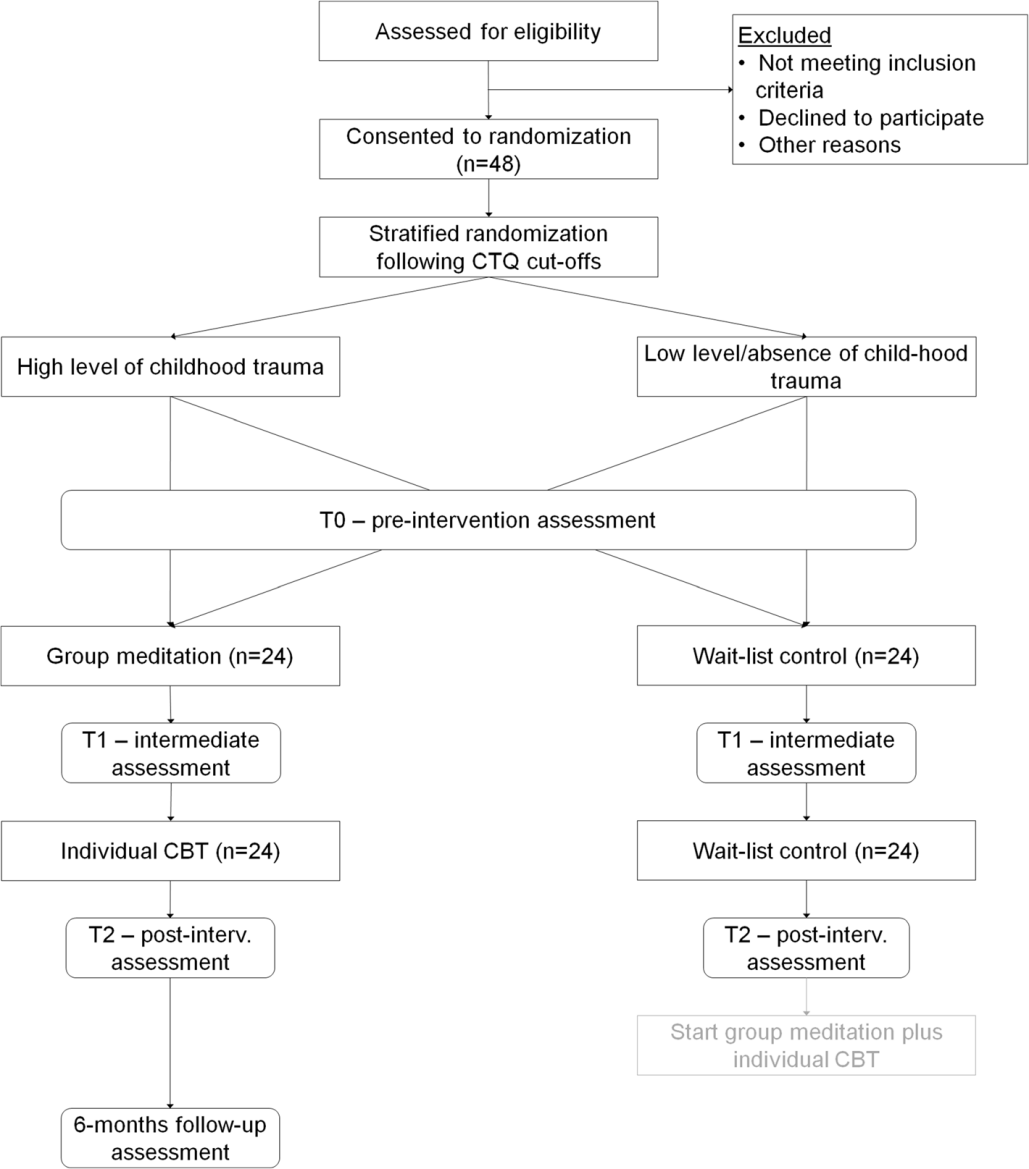
Flow diagram of the MeCBT study design. *CTQ* Childhood Trauma Questionnaire, *post-interv* post-intervention

The present study is a single-center randomized controlled trial of a combination of group meditation and individual CBT versus a wait-list control condition. Participants in the control group will receive no treatment or treatment as usual (e.g., antidepressants), but no psychotherapy during the treatment of the experimental group. A wait list control group has been included to control for unspecific expectation effects and symptom fluctuations. We strive for equal group sizes. Outcome measures will be assessed at four time points: before intervention (T0); after group meditation (T1); after individual CBT (T2); and at 6-month follow-up (T3). T3 will be assessed only in the experimental group because the waitlist condition terminates after T2. The wait list control group will be offered the MeCBT program upon completion of the waiting period and the T2 measurement. Due to this design of the study, there will be no untreated control group at the T3 measurement after 52 weeks, and follow-up effects will thus be uncontrolled and modeled using data from intervention participants only. The study is conducted at the Department of Clinical Psychology and Psychotherapy at Goethe University Frankfurt. The study protocol adheres to the Standard Protocol Items: Recommendations for Interventional Trials (SPIRIT) checklist (Additional file 1). Figure 1 illustrates the study design.

### Participants

This trial will include patients who meet the following criteria.

Inclusion criteria:

- Primary diagnosis of persistent depressive disorder according to DSM-5
- Aged 18–70 years
- No current psychotherapeutic treatment
- Written consent to participate in the study

Exclusion criteria:

- Αcute suicidality
- Substance abuse or dependence syndrome within the past 3 months
- Psychotic disorders or symptoms of bipolar disorder
- Borderline personality disorder
- Organic mental disorder or serious physical illness

Concurrent psycho-pharmacological treatment is not an exclusion criterion. Patients continue to receive a pharmacological anti-depressant treatment if it is indicated and are encouraged to keep it constant. Changes in the type and dose of the medication are recorded and documented. The waiting period for the wait-list control group is comparable to the usual waiting time for patients in outpatient psychotherapy care in Germany.

Participants will be excluded from the study if:

- The participant requests it (withdrawal of consent for participation)
- Further participation is associated with risks to the mental condition of the patient
- The participant’s condition requires inpatient treatment

Participants may withdraw from the study for any reason at any time. The principal investigator also may withdraw participants from the study in order to protect their safety or if they are unwilling or unable to comply with required study procedures. If a patient’s condition requires further treatment after withdrawal from the study or after completion of the program, the patient will be offered psychotherapy at the Center for Psychotherapy of the Goethe University Frankfurt or alternatively will receive support to find appropriate counseling or health care.

### Sample size

Previous within-group design studies [35, 38] showed moderate to large pre-post effect strengths of the group meditation program. The expected effect size for wait-list controlled studies cannot be derived from within-group design studies. However, given the expectations that a wait-list control group of patients with chronic depression will not change over the waiting period, and that the additional individual treatment component may increase the efficacy of the group meditation treatment, we assume at least a moderate effect of f = 0.25 in comparison to the wait-list control group. A power analysis was computed using G-Power 3.1.9.2 [56], with repeated measures ANOVA (within–between interaction) with two measurement time points, a power of 0.80, a strict α error probability of 0.01, and a correlation among the repeatedly measured dimensions of r = 0.7 [57], resulting in a sample size of 34 (using effect size specification as in SPSS). Hence, including a supposed drop-out rate of 25%, at least 46 patients must be recruited for this study. In order to realize group treatments with traditional group sizes (8–12 participants), we decided to perform the trial with two rounds or *cohorts* with equal size, each with one treatment and one wait-list control group. During the first round of the trial, half of the participants (cohort 1) will be recruited, randomized, and receive treatment or be in the wait-list control group. This procedure will be repeated in the second round with the second half of the participants (cohort 2). As a result, we will have a total of four therapy groups (two times one treatment and one control group). Since we strive for balanced therapy group sizes, we will include 12 participants per therapy group, i.e., 4 × 12 = 48 participants in total.

We would like to mention that as a result of this procedure we will have a stratification variable “cohort”. Since this stratification variable will be included only for administrative reasons, we will not include it in our statistical models [58].

An additional power analysis with the same parameters as above but with three measurements (including intermediate measurements), four groups (including a two-level stratification variable), and a less strict α error probability of 0.05 resulted in a sample size of 28.

### Participant recruitment process

Study participants are recruited in the Frankfurt metropolitan region through the Center for Psychotherapy at the Goethe University Frankfurt, self-help groups, psychosocial counseling centers, flyers, and advertisements on websites. In addition, we will recruit participants via press releases. Interested people will complete a brief telephone eligibility screening interview. Inclusion/exclusion criteria will be assessed by trained, independent clinicians using SCID-I and II [59, 60] and the Psychiatric Status Rating for Chronic Depression (PSR-CD) [61]. Since we intend to include a total of 48 participants and have two cohorts, for each cohort a total of 24 participants will be recruited for the trial.

### Randomization

Participants will be randomly and uniformly allocated to two test conditions. We suppose that differences in the individual history of childhood maltreatment may have an influence on the effectiveness of the treatment. Therefore, to prevent imbalances between treatment groups, the sample will be stratified based on the two-level-factor “reported adverse childhood experience” as measured before randomization by the Childhood Trauma Questionnaire [62]. Since we pursue the aim to inform participants about their allocation as soon as possible (see below), we decided to perform stratification and randomization as soon as half of the participants of one cohort (or one subcohort) have been enrolled. Thus, once 12 participants have been enrolled (subcohort 1 of 2), they are assigned to a stratum depending on their CTQ scores (“high level of childhood trauma/adversity” or “absence or low level of childhood trauma/adversity”). The CTQ cut-off values are set in advance. If odd numbers of participants are in the strata, the participant nearest the CTQ cut-off value will be assigned to the other stratum, despite not meeting the criteria for it. This procedure allows us to use random permuted blocks of even length (*n* = 2, 4, 6, 8, 10, or 12) in the strata. These steps will be repeated with subcohort 2 (of 2). Hence, within each of these strata, participants will be randomly assigned to the treatment or to the control condition, producing equal therapy group sizes.

We will send anonymized participant codes via e-mail to an independent statistician, who will conduct randomization. Randomization will be carried out with computer-generated random lists created using Microsoft Excel®.

### Blinding

Diagnosticians collecting observer rated measures will be blind to participant assignment as timing of assessments will be the same for all participants of a cohort. Blinding of diagnosticians should be maintained under all circumstances. To ensure that blinding is maintained, participants will be asked not to indicate their allocation status to the diagnosticians. Due to the design of the study, intervention blinding of participants and research workers, who also act as therapists, is not possible. The principal investigator is not blinded as he also implements the group therapies and individual therapies. Statistical analyses will be conducted by the authors, who will not be blinded or independent.

### Interventions

In our study, two approaches are to be combined in an approximately 4-month treatment program: a metta-based group meditation program focusing on the meditation of mindfulness and metta (eight sessions of 120 min each + one half-day retreat), and an individual CBT program (eight sessions of 100 min each) based on CBT and schema therapy that emphasizes the transfer of the meditation program into daily life. The metta-based group treatment has been tested in a pilot study and has been slightly modified for this trial. An individual subsequent CBT treatment including behavioral activation, cognitive restructuring and—optionally—elements from schema therapy has been added to this trial. The traditional concept of behavioral activation [63] was adapted to an interpersonal focus emphasizing self-confidence, positive social impact, and social interactions. Positive interpersonal behaviors are promoted based on personal values [64], which helps to create a more positive attitude to oneself and others in concrete actions. The intervention also involves the development of an accepting attitude towards negative emotions and cognitions [65], as well as actions involving benevolence to self and others [29]. The practice of combining these interventions has been done informally by our work group in several cases of chronically depressed patients from our pilot studies [35, 38] who had completed the studies but required additional treatment after termination of the study.

The mindfulness exercises are based on the manual of Segal, Williams, and Teasdale [66] and include a body scan exercise, sitting meditation, and the breathing space exercise. A shortened sitting meditation form is the basis of the metta meditation and the starting point of the meditation exercises. The breathing space exercise is used especially in stressful everyday situations for a more conscious perception of thoughts, feelings, and body sensations. It will also be used to help participants distance themselves from rumination. To achieve maximum acceptance among all participants, esoteric terms were avoided in the instructions where possible.

The exercises for metta meditation are based on the manual of Kearney et al. [67] using a German adaptation (Stangier U, Mendes A: Achtsamkeitsbasierte Loving Kindness Meditation, unpublished). The main aim is to focus on the perception of positive attitudes and feelings towards oneself and others. To elicit these positive emotions, kind wishes like “may (he/she/they/we/I) be safe/happy/live with ease/be free from suffering” are directed towards different recipients [68]. Starting with oneself, the formulas are gradually extended to a friend, a neutral person, a person one has difficulties with, all four together, and finally to all human beings. In the group meditation program, the selection of the recipient and the formulas are individualized according to personal relevance. If participants experience difficulties with meditation—which is a natural part of it—they are instructed to develop a mindful (i.e., non-judgmental and observing) attitude towards negative thoughts and feelings related to the present moment. In addition, there are tasks related to reflection and discussions about benevolence, which generally have proven to intensify the motivation to behave benevolently towards others [69]. The program also includes homework: daily meditation (at least once per day) and practice of benevolent behaviors, which shall be identified according to individual relevance, towards others and self. Participants receive audio recordings of guided meditations for their meditation homework. This should facilitate the correct learning of the meditation and ensure a standardization of this intervention component. Group treatment will be carried out by an approved psychotherapist and two clinical psychologists who are at an advanced stage of their post-graduate training in CBT.

The individual CBT following the group program is based on a manual for behavioral activation for depression [65] and schema therapy [54], both being adapted to the goals of the preceding meditation program.

The individual treatment part of the present trial will build on the experience gained from a randomized controlled clinical trial with patients with recurrent depression [70]. In this previous study, an approach of combining individual setting elements of MBCT as well as Acceptance and Commitment Therapy (ACT) with activity scheduling and other behavioral techniques was applied [71]. If patients report adverse memories about their childhood or other distressing events arising from dysfunctional schemas, techniques from schema therapy are used (e.g., schema diary, imagery rescripting, and empty chair dialogues).

Individual treatment will be carried out by two approved, experienced therapists and three clinical psychologists who are at an advanced stage of their post-graduate training in CBT. All therapists have received training in the treatment manual.

The participants will also be asked to respond daily to short electronic questionnaires throughout the project period. The questionnaire asks about the mood, whether the participants have meditated and, if so, how long and how well it worked. For this, text messages are sent with web links to the questionnaires. This has the function of a reminder but also of a meditation diary. In addition, these questionnaires will be evaluated weekly and give the study team and the therapist feedback on how the participants are adhering to the treatment. The questionnaires are located on the “unipark” platform and participants are asked to use their individual anonymized code to ensure protection of private participant data.

### Outcome measures

Proposed psychological outcome measures are as follows.

#### Primary outcome measure

- Severity of depressive symptoms as measured by the Quick Inventory of Depressive Symptomatology (QIDS-C) [72]

#### Secondary outcome measures

Observer rated:

- Emotion regulation as measured by the Operationalized Skills Assessment Inventory (German: “Interview zur Operationalisierten Fertigkeitsdiagnostik”; OFD) [73]

Self-report:

- Severity of depressive symptoms as measured by the Beck Depression Inventory II (BDI-II) [74]
- Behavioral and cognitive avoidance as measured by the Behavioral Activation for Depression Scale (BADS) [75]
- Emotion regulation as measured by the Affective Style Questionnaire (ASQ) [76]
- Benevolence/compassionate love as measured by the Compassionate Love Scale (CLS) [77], German version (Stangier U: Die Compassionate Love Scale – deutschsprachige Adaption, unpublished)
- Social pain as measured by the Social Pain Questionnaire (Schneider A, Schweitzer C, Stangier U: Fragebogen zu Sozialem Schmerz (FSS), unpublished)
- Mindfulness (including acceptance of negative thoughts) as measured by the Five Facet Mindfulness Questionnaire (FFMQ) [78, 79]
- Rumination as measured by the Response Styles Questionnaire, German version (RSQ-D) [80]
- Social motivation as measured by the Social Adaptation Self-evaluation Scale, German version (SASS) [81, 82]
- Interpersonal connectedness as measured by the Inclusion of Other in the Self Scale (IOS) [83]

We pursue three partially conflicting aims regarding randomization and baseline measurements of outcome variables:

1. Baseline measurements should take place as close as possible before the beginning of the group program in order to minimize any possible confounding with other effects.
2. Baseline measurements should take place before randomization, so that the knowledge about the allocation does not influence the participants.
3. Participants should know as soon as possible what condition they are allocated to in order to be able to set up the time for the program and make private plans, especially since the recruitment phase lasts over 3 months. If participants learn at short notice what condition they are assigned to, some participants might not be able to set up the time for the group program and hence drop out.

To comply with aims 1 and 2 (baseline measurement after randomization and as close to the beginning of the group as possible), we would have to perform the randomization shortly before the start of the group program. We decided to give aim 3 more weight and thus drop aim 2. As a result, after the randomization and before the baseline measurement participants are aware of their allocation. Knowing their allocation may motivate participants to report better or worse scores in the baseline outcome measures. We note this is not ideal and will discuss it in the primary trial publication.

Time points of outcome measures will be anchored to baseline measures, which take place within 2 weeks before the beginning of the group treatment. All further outcome measures (i.e., T1, T2, and T3) will take place at fixed time points after baseline measures, as defined in Table 1, i.e., 9, 26, and 52 weeks after baseline measures. 
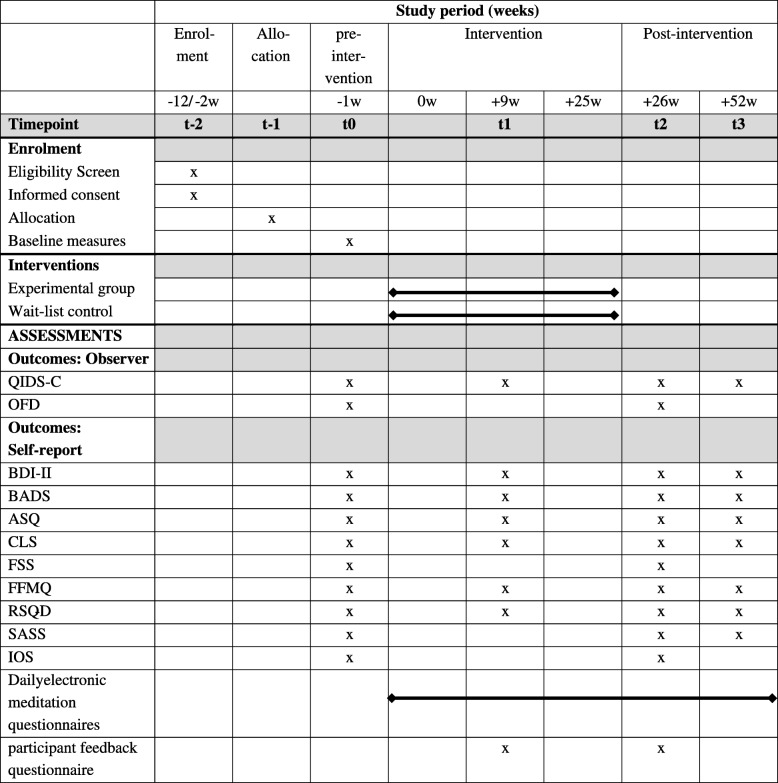

**Table 1:**
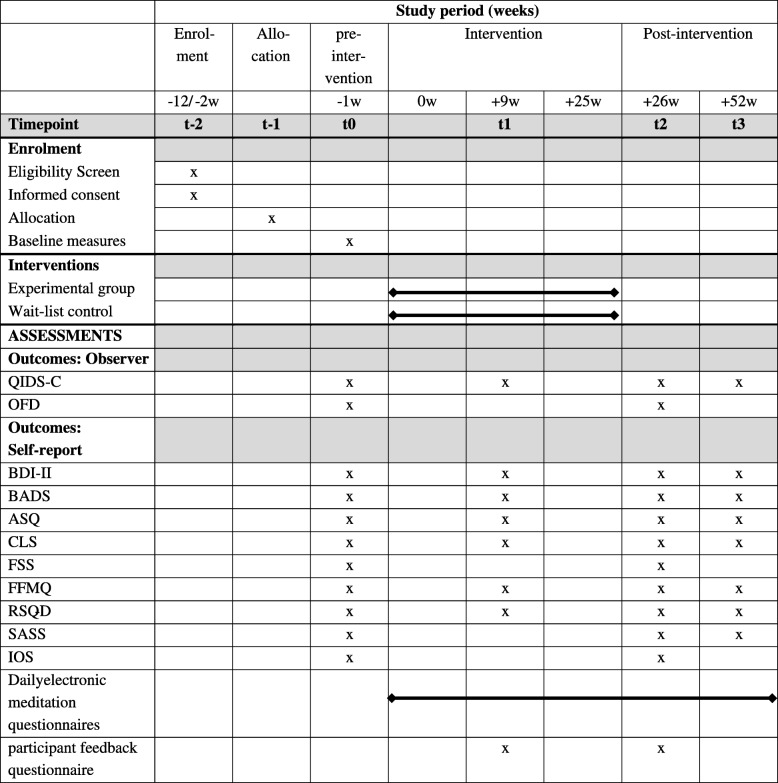
Standard Protocol Items: Recommendation for Interventional Trials (SPIRIT) diagram of assessments at enrolment, allocation, post-intervention, and 6-month-follow-up time points

*Abbreviations: ASQ* Affective Style Questionnaire, *BADS* Behavioral Activation for Depression Scale, *BDI*-*II* Beck Depression Inventory II, *CLS* Compassionate Love Scale, *FFMQ* Five Facet Mindfulness Questionnaire, *FSS* Social Pain Questionnaire, *IOS* Inclusion of Other in the Self Scale, *OFD* Operationalized Skills Assessment Inventory, *QIDS*-*C* Quick Inventory of Depressive Symptomatology, *RSQ*-*D* Response Styles Questionnaire, *SASS* Social Adaptation Self-evaluation Scale

The schedule of enrolment, interventions, and assessments is provided in Table 1.

### Data collection

Data from observer-rated measures will be collected by blinded, independent, trained clinical psychologists. The data will be logged on paper case report forms and afterwards independently entered by two project employees into a SPSS data sheet. All paper-based documents will be kept in a locked steel locker. Access to any data is restricted to trial personnel and investigators. The SPSS data sheet is stored on a secured server that is placed in a locked steel locker in a room accessible only by authorized IT personnel. The data values in the SPSS file are limited to appropriate ranges to ensure that only valid data are entered and will be checked for data entry mistakes.

Self-report measures, including all secondary outcomes except the OFD, will be collected by computerized questionnaires, using the academic online survey platform unipark [84]. The program ensures complete data entry. To ensure anonymity, participants will receive an individual identification code for completing the questionnaires, for data storage, and for statistical analysis. Identification codes are stored on hard copies accessible only to the study administrators. The data gathered via unipark will be downloaded and included in the final SPSS data file.

### Statistical analysis

The primary outcome measure is pre-post (i.e., T0–T2) changes on the QIDS-C. Intention-to-treat analyses will be employed in this trial. For the statistical analysis of the primary outcome we will use a three-factor mixed-design ANOVA with two two-level between-subjects factors (treatment and CTQ-based stratifier) and a two-level within-subjects factor (time, T0 and T2) in the model. For our primary outcome, the group difference at T2, we will use a model with an interaction of treatment by time. An additional analysis including T1 will be performed using planned contrasts to examine changes in depression after group and individual treatment. We will use a repeated measures MANOVA including a time by treatment interaction to assess the effects of secondary outcomes, which we assume to be moderately inter-correlated, followed by univariate repeated measures analyses.

An intended aim was to detect mechanisms of change, which requires a mediation analysis. However, mediation analyses using structural equation analyses and latent growth curve analyses require a larger sample size, more measurement occasions of potential mediators than given in our study, and the control of confounding variables between mediators and outcome [88]. Therefore, exploratory regression analyses using pre-post differences in primary and secondary outcome after the meditation group treatment and the individual treatment will be performed to detect possible candidates for mediation analyses within future studies. Missing data will be handled using multiple imputation procedures. Data collected about adverse events and serious adverse events will be summarized by type and frequency, and regression analyses will be conducted to explore relations between adverse events and treatment. For statistical analysis, the computer program SPSS® (Statistical Package for Social Sciences, version 25) will be used.

### Trial governance

The Trial Management Group consists of the principal investigator and the study team (two clinical psychologists and two graduate student research assistants) and will provide overall management of the study including set-up of the study, recruitment, training of independent clinical raters, providing treatment, and interpretation of results. Due to the small scale of our trial, no independent trials steering committee has been planned. Overall supervision of the trial is provided by the principal investigator. Weekly meetings of the project team are held and adherence to the study protocol is checked at regular intervals. Changes to the study protocol will be communicated to the funding source, the ethics committee, and the trial registry. No interim analysis is planned due to the small scale of the study. Therapists will be video-recorded during the group treatment sessions where participants give their consent. The treatment sessions will be supervised by the principal investigator in order to continuously ensure adherence to the study protocol. We will ask the therapists to give informal feedback and participants to give formal feedback on their experience and their acceptance of the treatment program via an anonymous paper-based questionnaire. The questionnaire consists of open-ended questions, which will be evaluated qualitatively.

### Plans to promote participant retention and complete follow-up

Once a participant is enrolled, the Trial Management Group will make every reasonable effort to keep in contact with the participant during the entire study period. If a participant expresses doubts about further participation, a personal dialogue will be offered to him or her to possibly find a way to continue participating in the study. In order to pursue participant retention during the entire study period, prior to data collection time points participants will be contacted and reminded of the upcoming data collection. No further measures (e.g., material or financial incentives) are planned to promote participant retention. If participants withdraw from treatment or are excluded from interventions due to risk or otherwise deviate from the study protocol, we will attempt to collect all planned outcome data. We will only refrain from collecting outcomes from those who withdraw fully from the entire trial.

### Adverse event monitoring

An adverse event is any untoward medical occurrence (e.g., a symptom or disorder) in a patient temporarily associated with our treatment. An adverse event does not necessarily have a causal relationship with the treatment. A serious adverse event is any untoward medical occurrence that results in death, is life-threatening, requires inpatient hospitalization, or results in persistent or significant disability/incapacity [85]. Adverse events and serious adverse events will be collected throughout the trial and will be reported regarding type and time of occurrence. Collected data will be summarized by type and frequency, and regression analyses will be conducted to explore relations between adverse events and treatment. All therapists will be informed about potential adverse effects of meditation and will be encouraged to be particularly sensitive to this issue. The participants will be informed about possible side effects of psychotherapy and of meditation, and are encouraged to report negative changes.

All therapists involved in the trial are under continuous supervision by the principal investigator and will report weekly on suicidal ideations of patients or possible increase of risk for suicidal actions. Somatic conditions are checked by physicians prior to the participation in the study. Risk of side effects is considered low, but potential treatment-related adverse events will be carefully monitored. Medication will be checked regularly by psychiatrists consulted by the participants in the trial who take psychopharmacological drugs. Patients participating in the trial are instructed to keep their medication and change only after consulting their psychiatrist.

In an emergency, immediate contact with stationary psychiatric facilities in the Frankfurt area can be made. Since the interventions will be carried out by clinical psychologists and will be supervised by an experienced therapist, any worsening of the mental health condition of the participants can be detected and met accordingly.

## Discussion

This study investigates the efficacy of MeCBT, a new, low-intensity, cost-effective intervention for PDD. Conventional treatment methods for depression have mostly focused on reducing negative affect and teaching interpersonal skills. MeCBT extends the focus of treatment to positive affect and benevolence towards other people. Previous studies have shown that stand-alone group meditation including mindfulness and metta is an appropriate technique for increasing positive affect in chronically depressed patients and reducing depressive symptoms. Through this, stand-alone group meditation is a cost-effective intervention to stimulate the beginning of the therapeutic process, but is presumably not sufficient for sustainable symptom reduction. The additional individual CBT offers effective treatment of specific issues and helps to implement meditation practice more permanently into everyday life, which may reduce the risk of relapse.

Another potential strength of MeCBT is that meditation may appeal to people concerned about stigma of seeking psychotherapy. This may facilitate the decision to seek treatment and avoid further persistency of depression. In addition, meditation as a “new” approach may be associated with positive expectations. Our trial also tests new digital technology in psychotherapy (e.g., daily meditation reminders via text message). Daily reminders as well as online meditation/mood protocols may not only support participants in practicing meditation but also allow for the empirical evaluation and monitoring of the current therapy process. Further analyses may help to identify possible candidates for mediation analyses within future studies.

While our trial is observer-blinded, and allocation is conducted by an independent statistician, participants and therapists are not blinded about the allocation. This may have an influence on patients’ conditions, especially in the wait-list control group. Results of this study will be interpreted taking the limitations of wait-list control designs [86, 87] into account. To prevent possible nocebo effects in the wait-list control group [87], participants in this group will be encouraged to continue their usual pharmacological treatment.

It will not be possible to differentiate between specific effects of MeCBT (group and individual phase) and common factors of psychotherapy. Nevertheless, it will be possible to approximately compare effect sizes of our trial to other RCTs using wait-list control groups and a homogenous group of patients with PDD. We will be able to compare the effects of the group therapy program and, after subsequent individual therapy, of the entire MeCBT-program to a wait-list control group. However, due to the design of our study (for instance, the treatment elements will have a fixed sequence) we will not be able to determine the specific influences of the two treatment components on the overall outcome, i.e., whether they are additive or multiplicative. This would require a different design.

A further limitation of our study is the restriction to PDD as primary treatment diagnosis. It does not consider persistent depressive symptoms in the context of bipolar disorder. There may be further possible challenges, especially regarding the acceptance of the treatment content by the patients and a continuous participation in our program. A new treatment method can raise hope, but disappointment could also occur quickly. Therefore, it will be important to meet the expectations of the participants and communicate the aims, advantages, and limitations of our treatment program transparently. A more general limitation is that our trial will only allow us to draw conclusions for a western population. Despite all limitations, we expect the results of our study to make an important contribution to refining and testing a treatment manual specifically tailored to PDD. If the results of this study support the efficacy of this treatment approach in lowering depression symptoms and this form of treatment is accepted by clinicians as well as patients, MeCBT could improve the psychosocial situation of many people affected by this disorder.

## Trial status

Research protocol, version 03, February 2018. The MeCBT study began recruitment on 2nd January 2018. The first treatments were implemented mid April 2018. The last participant was recruited on 4th April 2019, and the trial is expected to be completed in July 2020.

## Supplementary information

**Additional file 1.** SPIRIT 2013 Checklist: Recommended items to address in a clinical trial protocol and related documents.

## Data Availability

The study protocol is made publicly available through this publication. The main results are intended to be published in a high-impact peer-reviewed journal within 6 months after the trial end date (approximately 2020/21). Individual participant data will be available for investigators whose proposed use of the data has been approved by an independent review committee identified for this purpose. Data will be available beginning 6 months and ending 36 months following article publication. This includes individual participant data that underly the results, reported in articles that are yet to be published, after de-identification and in accordance with the European General Data Protection Regulation (GDPR). To gain access, researchers requesting data will need to sign a data access agreement. For information regarding submitting proposals and accessing data please contact the study sponsor.

## Abbreviations

ANCOVA: Analysis of covariance
ASQ: Affective Style Questionnaire
BADS: Behavioral Activation for Depression Scale
BDI-II: Beck Depression Inventory II
CBASP: Cognitive Behavioral Analysis System of Psychotherapy
CBT: Cognitive behavioral therapy
CD: Chronic depression
CLS: Compassionate Love Scale
CTQ: Childhood Trauma Questionnaire
DSM-5: Diagnostic and Statistical Manual of Mental Disorders, Fifth Edition
FFMQ: Five Facet Mindfulness Questionnaire
FSS: Social Pain Questionnaire (German: Fragebogen zu Sozialem Schmerz)
IOS: Inclusion of Other in the Self scale
IPT: Interpersonal psychotherapy
MBCT: Mindfulness-based cognitive therapy
OFD: Operationalized Skills Assessment Inventory (German: Interview zur Operationalisierten Fertigkeitsdiagnostik)
PDD: Persistent depressive disorder
QIDS-C: Quick Inventory of Depressive Symptomatology
RSQ-D: Response Styles Questionnaire, German version
SASS: Social Adaptation Self-evaluation Scale
SPIRIT: Standard Protocol Items: Recommendation for Interventional Trials
